# News and Events of the Week

**Published:** 1910-10-01

**Authors:** 


### NEWS AND EVENTS OF THE WEEK.

ON Monday, October 3, Field-Marshal Viscount Kitchener of Khartoum will distribute the prizes at the opening of the seventy-sixth winter session of the Middlesex Hospital Medical School, at 3 P.M.

MR. WILLIAM BERRY, F.R.C.S., of Park House, Wigan, Lancs, Medical Officer of Health for the County Borough of Wigan, who died on July 29, has left estate valued at £7,938 gross, and £6,276 net.

In his annual report on the trade of Swatow, the British Consul states : "Chinese missionaries mention the growing habit of morphia-injection among the natives as taking the place of opium, and it seems to be a fact that morphine can be purchased in the local shops more cheaply than it can be imported from Europe."

THE Court of Common Council, presided over by the Lord Mayor, passed the following rsolution last week : "That a scholarship be established at St. Thomas's Nursing Home in connection with the City of London Schools to perpetuate the memory of the late Miss Florence Nightingale, and that it be referred to the City of London Schools Committee to consider and report as to the best means of establishing a scholarship, with power to confer with the Coal, Corn, and Finance Committee thereon."

THE Berlin police regulations now demand that taxi-cab drivers should submit themselves to medical examination before they can be granted a licence. The examination makes special investigations into eyesight and hearing and certain nervous defects.

MME. PASTEUR, the widow of the great French bacteriologist, has died at Arbois, in the Jura. Mme. Pasteur, who was in her eighty-fifth year, married M. Pasteur in 1850, and his "Life" by M. Valéry Radot, shows how his achievements were facilitated by the ideal home which she made for him.

JEAN DES GALERES, an escaped galley-slave, and the hero of a French novel bearing that title, written by Edgar Monteuil, and published in the seventies, was given an *ordonnance* by one of his accomplices, a doctor, for hyoscine, for the purpose of poisoning a banker, whose premises he had planned to rob. The pharmacien, says a writer in the *Chemist and Druggist*, gave him a less potent drug, which only put his victim to sleep temporarily. In another novel, "A Wingless Angel," written by J. E. Muddock, published in 1878, and dedicated to Dr. Benjamin Ward Richardson, hyoscyamia was the poison used to remove one of the charact[illegible]. Curiously, the name of the heroine of this novel was Belmore.

The opening of the session of the School of Pharmacy, Pharmaceutical Society of Great Britain, will take place at 17 Bloomsbury Square, London, W.C., on October 5, at 3 p.m. The address will be delivered by Mr. David Howard, and the Pereira Medal will be presented by the President. At the conclusion of the meeting the subscription portrait of Mr. Walter Hills will be formally presented.

The longevity popularly credited to Brighton is borne witness to by the Rev. J. P. Bacon Phillips, Rector of Crowhurst, who in visiting the infirmary attached to the workhouse at Brighton found three old women: "Mrs. Norman, the centenarian daughter of a centenarian, will, all being well, be 101 years of age on November 5. Mrs. Sinden, who walks about the ward every day, the mother of eleven children, and with an excellent memory, will be 101 if she lives until February 28; and Mrs. Dewey, 97 years of age, the mother of ten children."

Queen Alexandra has been pleased to make the following among other additional appointments in her Majesty's Household, to be dated May 7, 1910: To be Physician in Ordinary and Surgeon Apothecary to the Household, Sir Francis Henry Laking, Bart., G.C.V.O., K.C.B.; to be Surgeon in Ordinary, Sir Frederick Treves, Bart., G.C.V.O., C.B.; to be Physician Extraordinary and Surgeon Apothecary at Sandringham, Sir Alan Reeve Manby, M.V.O.; to be Laryngologist, Milsom Rees, Esq., F.R.C.S.Ed.

An Association of Inspectors of Midwives has been formed, and held its first meeting last Saturday at the Midwives' Institute. The number of lady inspectors of midwives, appointed by county or borough councils, is steadily increasing, and their work is recognised as a powerful factor in the education and professional improvement of midwives, especially those who obtained their certificates under the *bona-fide* clause. The objects of the association include the consideration and discussion of the best methods of bringing more uniformity into the administration of the Act, and of obtaining trustworthy and prompt information from the midwives themselves. As regards the new Midwives Bill, which passed the House of Lords last session, the association pledges itself to endeavour to obtain the amendment or omission of certain clauses which they deem prejudicial. Dr. Macrory, senior inspector for London, was elected President, and all information can be obtained from the Hon. Secretary, Miss du Sautoy, 16 Elm Grove, Taunton.

Professor Osler's "Principles and Practice of Medicine" has been translated into Chinese by Dr. P. B. Cousland, President of the China Medical Missionary Association, Shanghai. It is the only first-class work on the principles and practice of medicine that has so far been translated into Chinese. Other translation work is in progress. Dr. Cochrane, of Peking, is translating Heath's "Anatomy"; Dr. McAll, of Han-kau, Stengel's "Pathology"; Dr. Cormack, Peking, Hutchison and Rainy's "Clinical Methods"; and Dr. Neal, of Tsi-nan, is engaged upon Fuch's "Ophthalmology." A new and compact "Systematic Anatomy" is passing through the press. An atlas of beautiful anatomical plates has just been printed for the China Medical Missionary Association by the Oxford Press and Messrs. Hodder and Stoughton, at a cost, apart from the letterpress, of £500, part of which has been contributed by the China Emergency Appeal Committee. As dissections of the human body are not yet allowed in China, such plates are of great importance.

Two new health caravans are to be sent on tour immediately by the Women's Imperial Health Association of Great Britain, one through the Eastern Counties and the other in the London parks. This step has become necessary in consequence of the great number of applications which were received by the Association for visits from the "Aurora," the first health caravan which recently commenced at Maidenhead its mission of carrying a health propaganda broadcast through the land. Besides the organised meetings garden-parties and other social functions have been attended by the caravan, and in several places active measures are being taken by local ladies to form district branches of the Association, the caravan itself in one instance being used for holding the preliminary committee meeting. Inquiries as to caravans, literature, and requests for information as to branches have been pouring in from all parts of the United Kingdom, and even so far afield as Australia, South Africa, and Italy.

The London County Council has given permission for the health lectures, illustrated by cinematograph pictures, to be given in Finsbury, Battersea, and Victoria Parks. This is the first time that the L.C.C. have sanctioned a cinematograph display in the London parks, and the occasion will be doubly interesting, because it marks an important development in the history of cinematograph displays. The lectures will be given during the day and an invention for daylight displays of cinematograph pictures will be used for the first time.

The Eastern Counties Caravan of the Women's Imperial Health Association of Great Britain will be dedicated at the Royal Botanic Gardens, Regent's Park, by Muriel Viscountess Helmsley, the President of the Association, on Saturday, October 1, 1910. The caravan, which will be known as the "Florence Nightingale," will be entirely manned by women, and a fully-qualified woman doctor will deliver the lectures. The itinerary will include: Romford, Brentwood, Chelmsford, Witham, Colchester, Manningtree, Ipswich, Haughley, Diss, Norwich.

The following are the arrangements for next week at the Medical Graduates' College and Polyclinic, 22 Chenies Street, W.C.: Monday, October 3, at 4 p.m., Dr. Wilfred Fox, Clinique (Skin); at 5.15 p.m., Dr. Robert Jones, "The Teaching of Insanity in the British Isles." Tuesday, at 4 p.m., Dr. Harry Campbell, Clinique (Med.); at 5.15 p.m., Dr. Scanes, "The Causation and Cure of Obstinate Nasal Discharges." Wednesday, at 4 p.m., Mr. A. H. Tubby, Clinique (Surg.); at 5.15 p.m., Mr. Richard Lake, "Eustachian Obstruction." Thursday, at 4 p.m., Mr. E. Laming Evans, Clinique (Surg.); at 5.15 p.m., Dr. Dundas Grant, "Some Practical Points in the Diagnosis and Treatment of Tuberculosis of the Larynx." Friday, at 4 p.m., Mr. E. Kenneth Campbell, Clinique (Eye).

